# Supplementary material for: Wiki Surveys: Open and Quantifiable Social Data Collection
Source: PLoS One. 2015 May 20;10(5):e0123483. doi: 10.1371/journal.pone.0123483 (PMC4439069; doi:10.1371/journal.pone.0123483)
Supplement: S1 Text — (PDF) [file pone.0123483.s001.pdf]

SI 1: Website implementation

In this SI, we describe the procedures used for data collection on our website, `www.allourideas.org`. When implementing pairwise wiki surveys on the website, we encountered three main methodological issues: 1) choosing pairs to present to respondents; 2) using the responses to estimate the score; and 3) ensuring data quality. In all cases, we solved these problems using relatively simple heuristic approaches. Our heuristic for score estimation on the website differs from the technique used in the data analysis section of the paper; we present it here for completeness. We are confident that many of these approaches will be improved based on future research.

1 Selection of pairs

The simplest way to select pairs for the respondents would be to sample with uniform probability from the set of pairs. However, because pairwise wiki surveys are collaborative, respondents contribute new items throughout the process, which means that pairs with user-contributed items will tend to have fewer responses. Therefore, the simple approach would result in more responses—and therefore more precise estimates—for seed items than user-contributed items. This disparity is problematic because the user-contributed items are potentially the most interesting. Instead, it is preferable to spread the responses more evenly over the set of pairs. Therefore, we developed a "catch up" algorithm, which shows pairs with fewer completed responses with higher probability. In essence, it helps newer pairs "catch up" to older ones in terms of number of responses. Specifically, the draw-wise probability for a given pair (i, j) is:

$$p_{i,j} = \frac{min\left(\frac{\frac{1}{(n_{i,j}+1)^{\alpha}}}{c_1}, \tau\right)}{c_2} \tag{1}$$

where $n_{i,j}$ is the number of votes on prompt (i, j), α is a parameter that weights the number of responses, and τ is a "throttle" to ensure that the draw-wise probability never exceeds some threshold (it could create a poor user experience if the same pair had a draw-wise probability of, say, 0.5). Finally, c_1 and c_2 are normalizing constants to ensure that the distribution sums to 1.[1] Although somewhat awkward-looking, Eq. 1 is straightforward to implement and runs very quickly. As a first step we choose $\alpha = 1$ and $\tau = 0.05$, but the optimal values of these parameters are an open question.

2 Estimating score

We decided to make the score of each item available to all respondents in real time. This requirement for real time calculation made it impossible for us to use the statistical methods described in the data analysis section of this paper. Therefore, for the website, we developed a simpler method of estimating the score. In the cases considered in the paper, the two estimates of the score were very similar; there was a correlation of about 0.95 in both cases.

Recall that the score of an item is the probability that the item will beat a randomly chosen item for a randomly chosen respondent. Given this focus on the probability of a win, we choose a binomial model. If one assumes a uniform prior for a binomial random variable, the resulting posterior for the probability of a win follows a Beta distribution [1]. If we multiply the expected value of that Beta distribution by 100 (to place things on a more natural scale), we have

$$\widehat{s_i^{\prime}} = \frac{(w_i + 1)}{(w_i + 1) + (l_i + 1)} \times 100 \tag{2}$$

where w_i is the number of wins for item i and l_i is the number of losses for item i; see [1, Ch. 3] for a derivation. Thus, the estimated score ranges from 0 to 100 and resembles a simple winning percentage with an additional term that provides some smoothing.

This approach is both easy to calculate and reasonably principled because it is derived from standard Bayesian methods. It also has several desirable practical properties including that it produces a reasonable estimate for new items that have not appeared ($\widehat{s_i^{\prime}} = 50$) and the amount the

[1]The normalizing constants are $c_1 = \sum_i \frac{1}{(n_{i,j}+1)^{\alpha}}$ and $c_2 = \sum_i min\left(\frac{\frac{1}{(n_{i,j}+1)^{\alpha}}}{c_1}, \tau\right)$ where τ is the throttle, the maximum probability for a pair appearing in a draw.

score changes with any specific vote decreases as the number of votes on the item increase. However, the approach also has some limitations. First, it does not account for the fact that responses are nested within respondents. In other words, a respondent who contributes 100 responses will have 100 times the influence as someone who responds only once. Also, this approach does not consider the "strength of schedule" (i.e., the scores of the items that a given item has competed against). For example, this scoring approach gives equal weight to an item beating a popular item as to one beating an unpopular item. For these reasons and others, we developed the model described in the data analysis section of this paper, which does not suffer from these two limitations, but which takes many hours to compute.

3 Data quality issues

In all data collection, researchers must be wary of respondents who wish to manipulate results, but those risks are particularly salient in this research. In order to make our results more manipulation-resistant [2], we flagged some responses as invalid. These invalid responses were collected, but not included in the final analysis. There are two ways that a response can be flagged as invalid. First, if we receive multiple, consecutive responses for the same pair (as would occur if the respondent tried to click several times before the page reloads), then only the first response is marked valid; the others are marked invalid and are not included in the data files generated by `www.allourideas.org`. Second, all responses that occur immediately following the response "I can't decide" are marked invalid but are still included in the data files generated by `www.allourideas.org`. These responses are not included in estimation because in a previous pairwise wiki survey we detected a respondent who attempted to manipulate the results by clicking "I can't decide" until his or her preferred idea was presented, at which point he or she voted for that idea. Our flagging procedure prevents this manipulation from influencing the results. Though our approach probably invalidates some legitimate data, we prefer to err on the side of caution. Finally, we note that these procedures do not protect against all possible forms of manipulation, and future research will be necessary to make wiki surveys more manipulation-resistant. In the two case studies presented in this paper, we do not believe that any large-scale manipulations took place.

A second potential data quality issue arose because our pairwise wiki survey website had no

login system to verify a respondent's identity. We decided not to create such a system because we wanted to minimize barriers that might create differential non-response. However, the lack of authentication means that there is no guarantee that each of our respondents is unique. Each participant is defined by a "session" at the website, and a session is created when a browser that is not currently in a session visits the site. If there are 10 minutes of inactivity on the site, the current session is terminated; future activity on the site would result in a new session being created. The sessions are tracked with browser cookies. There are many ways that a single person could create multiple sessions and thus be considered multiple respondents (e.g., by visiting the wiki survey from a new browser or by deleting cookies). We do not believe that a single person creating multiple sessions caused any large-scale problems in the two case studies presented in this paper.

References

1. Hoff PD. A First Course in Bayesian Statistical Methods. 2nd ed. Dordrecht: Springer; 2009.

2. Resnick P, Sami R. The Information Cost of Manipulation-Resistance in Recommender Systems. In: Proceedings of the 2008 ACM Conference on Recommender Systems. RecSys '08; 2008. p. 147–154.
