# Supplementary material for: Wiki Surveys: Open and Quantifiable Social Data Collection
Source: PLoS One. 2015 May 20;10(5):e0123483. doi: 10.1371/journal.pone.0123483 (PMC4439069; doi:10.1371/journal.pone.0123483)
Supplement: S2 Text — (PDF) [file pone.0123483.s002.pdf]

SI 2: Data analysis

1 Statistical model

As described in the paper, a main statistical challenge is to use the responses (e.g., Table 1) to estimate the opinion matrix, $\boldsymbol{\Theta}$, which represents how much each respondent values each item. To do this, we begin by assuming a model for how the votes are generated; a natural first choice would be

$$Pr(a \text{ beats } b \text{ in session } j) = F(\theta_{j,a} - \theta_{j,b}) \tag{1}$$

where $\theta_{j,a}$ is the amount that respondent j values item a. That is, the probability that item a beats item b is a function of the difference in the appeals of the two items $\theta_{j,a}$ and $\theta_{j,b}$. In previous work, numerous functional forms have been assumed for F, but the two common choices are the cumulative standard normal—resulting in the Thurstone-Mosteller model [1, 2]—or the logistic function—leading to the Bradley-Terry model [3]. In fact, Stern [4] has shown that the Thurstone-Mosteller model and the Bradley-Terry model can both be viewed as special cases of a more general model, and empirically, both models produce estimates that are essentially equivalent [5]. However, the Thurstone-Mosteller model is much easier to work with computationally because it facilitates the Gibbs sampling updates as described below. For that reason we assume that

$$Pr(a \text{ beats } b \text{ in session } j) = \Phi(\theta_{j,a} - \theta_{j,b}) \tag{2}$$

where Φ is the cumulative standard normal distribution. Thus, we map the difference between the appeals, which ranges from $-\infty$ to ∞, to a value that ranges from 0 to 1. Future work could explore the robustness of our estimates to the choice of the standard normal or could attempt to estimate the shape of F directly. Another extension of the model would allow for "I can't decide"

Respondent	Response	Pair	
1	1	[**item 1**]	item 4
1	2	item 3	[**item 1**]
1	3	[**item 4**]	item 3
2	4	[**item 3**]	item 4
2	5	item 4	[**item 2**]

Table 1: An example of five responses given by two respondents. The bolded item is the one that was chosen by the respondent.

responses, which are not included in our current modeling framework.

Given the response model described in equation 2 and assuming that responses are independent, we can create a design matrix $\boldsymbol{X}$ and outcome vector $\boldsymbol{Y}$ so that the likelihood can be written to resemble a standard probit model,

$$p(\boldsymbol{\theta} \mid \boldsymbol{Y}, \boldsymbol{X}) = \prod_{i=1}^{V} \Phi(\boldsymbol{x}_i^T \boldsymbol{\theta})^{y_i} (1 - \Phi(\boldsymbol{x}_i^T \boldsymbol{\theta}))^{1-y_i}. \tag{3}$$

In this case, $\boldsymbol{X}$ has V rows and $J \times K$ columns, where V is the number of votes, J is the number of respondents, and K is the number of items. Therefore, $\boldsymbol{x}_i = (x_{i1}, x_{i2}, \ldots x_{im})$ and $m = J \times K$. In order for the algebra to work out properly, each row in $\boldsymbol{X}$ has a "1" in the column of the respondent/item that appeared on the left of the pair and a "-1" on the column of the respondent/item that appeared on the right of the pair. $\boldsymbol{Y}$ is a vector with V entries that has a "1" if the item on the left is chosen and "0" if the item on the right is chosen. For example, the votes in Table 1 would lead to

$$\boldsymbol{Y} = \begin{pmatrix} 1 \\ 0 \\ 1 \\ 1 \\ 0 \end{pmatrix} \quad \text{and} \quad \boldsymbol{X} = \begin{array}{c} \begin{array}{cccccccc} \theta_{1,1} & \theta_{1,2} & \theta_{1,3} & \theta_{1,4} & \theta_{2,1} & \theta_{2,2} & \theta_{2,3} & \theta_{2,4} \end{array} \\ \left(\begin{array}{cccc|cccc} 1 & 0 & 0 & -1 & & & & \\ -1 & 0 & 1 & 0 & & & 0 & \\ 0 & 0 & -1 & 1 & & & & \\ \hline & & & & 0 & 0 & 1 & -1 \\ & 0 & & & 0 & -1 & 0 & 1 \end{array}\right) \end{array}$$

By explicitly attempting to estimate each respondent's opinion about each item, this modeling approach allows for heterogeneity in the preferences of the respondents. However, the cost of such flexibility is that there are an enormous number of parameters to be estimated; in each of the

case studies in the paper, there were about 375,000 parameters to estimate (~1,500 respondents × ~250 items). Therefore, to add more structure to the problem and to allow for partial pooling of information across respondents [6, 7], we add hierarchical terms in the model that assume that the opinions about each item are normally distributed with an item-specific mean μ_k and a common standard deviation of σ ($\theta_{\cdot,k} \sim N(\mu_k, \sigma)$),

$$p(\boldsymbol{\theta} \mid \boldsymbol{Y}, \boldsymbol{X}, \boldsymbol{\mu}, \sigma) = \prod_{i=1}^{V} \Phi(\boldsymbol{x}_i^T \boldsymbol{\theta})^{y_i} (1 - \Phi(\boldsymbol{x}_i^T \boldsymbol{\theta}))^{1-y_i} \times \prod_{j=1}^{J} \prod_{k=1}^{K} N(\theta_{j,k} \mid \mu_k, \sigma) \tag{4}$$

where $\boldsymbol{\mu} = \mu_1 \ldots \mu_K$ and σ is assumed to be 1. In the case studies considered in this paper, we re-ran the model with $\sigma = 0.5$ and $\sigma = 2$ as a robustness check, and in both cases, the results were essentially the same as when $\sigma = 1$. Future work could improve the model by estimating a σ_k for each item or even estimating the functional form that the $\theta_{j,k}$ follow for each k.

Finally we add conjugate priors to yield the following posterior distribution:

$$\begin{aligned} p(\boldsymbol{\theta}, \boldsymbol{\mu}, \mid \boldsymbol{Y}, \boldsymbol{X}, \sigma, \boldsymbol{\mu_0}, \boldsymbol{\tau_0^2}) \quad \propto \quad & \prod_{i=1}^{V} \Phi(\boldsymbol{x}_i^T \boldsymbol{\theta})^{y_i} (1 - \Phi(\boldsymbol{x}_i^T \boldsymbol{\theta}))^{1-y_i} \times \prod_{j=1}^{J} \prod_{k=1}^{K} N(\theta_{j,k} \mid \mu_k, \sigma) \\ & \times \prod_{k=1}^{K} N(\mu_k \mid \mu_{0[k]}, \tau_{0[k]}^2) \end{aligned} \tag{5}$$

As is common in discrete-choice models [8], the model above is only weakly identified because one could add a constant c to all the θ parameters and leave the posterior largely unchanged (it may be easier to see this non-identifiability from the model for a single response (Eq. 2)). Therefore, we pick an arbitrary item to have $\mu_k = 0$ which requires setting the hyper-parameters $\mu_{0[k]} = 0$ and $\tau_{0[k]}^2 = 0.000001$. For the remaining items, we set weakly informative priors: $\mu_{0[k]} = 0$, $\tau_{0[k]}^2 = 4$. For readers accustomed to graphical models, our model for the 5 responses in Table 1 is presented in Figure 1.

This model is just one possible model for estimating the opinion matrix from responses. Further, we do not yet have good procedures for testing modeling assumptions, and we do not know how robust the model is to violations of underlying assumptions. We suspect that the biggest problems will arise from our assumption about the distribution of opinions across respondents. In the pairwise wiki surveys analyzed in this paper, it is important to realize that many respondents did not

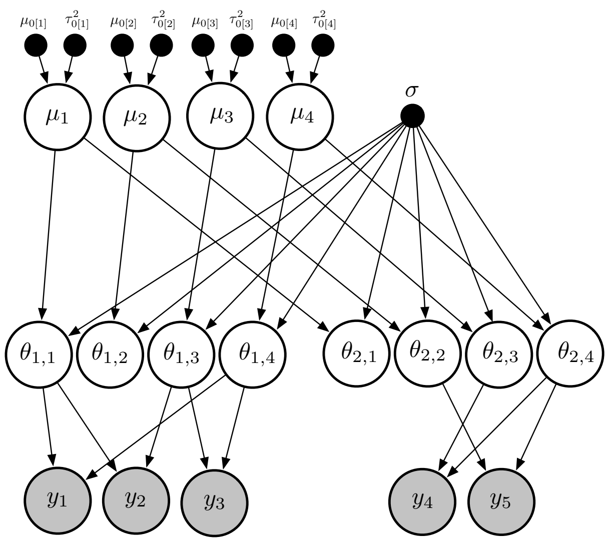

Figure 1: Graphical representation of the model (see Equation 5). This graphical model shows the assumed data generating process for the sample data shown in Table 1. At the top of the figure, priors are used to generate item-specific means (e.g., μ_1). Next, these parameters and σ, which is assumed to be 1, generate the elements of the opinion matrix (e.g., $\theta_{1,1}$). Finally, these elements of the opinion matrix generate the observed responses (e.g., y_1). The challenge is then to estimate the unknown parameters ($\boldsymbol{\theta}$ and $\boldsymbol{\mu}$) from the observed data.

encounter many of the items. Thus, there are actually two types of θ parameters, those that are informed by a specific vote ($\boldsymbol{\theta_v}$) and those that are not ($\boldsymbol{\theta_h}$). We exploit this feature of the data later when describing our approach to computation, but it also has important substantive implications. Our hierarchical modeling assumption means that we are assuming that the θ's we estimate based on a specific vote are directly informative of the θ's for which we have no specific vote (and therefore must make an estimate using data from other respondents). We can think of two cases in which this assumption might be unreasonable. First, consider an item uploaded by respondent j. All respondents before j did not have a chance to respond to this item so we will estimate their opinions about the item based on the respondents after j. Therefore, if for some reason the preferences of respondents vary systematically over time, our procedure will not work well. Second, the greedy nature of the wiki survey could also lead to problems if people who respond many times have systematically different preferences than those who respond fewer times. For example, imagine that there are two types of people: vegans and non-vegans. Further, imagine that all vegans love bicycles, all non-vegans hate bicycles, and that vegans contribute more responses than non-vegans. Now, if we have a respondent j that did not encounter an idea k ("more

bike racks in Manhattan"), the model will estimate $\theta_{j,k}$ based on the other $\theta_{\cdot,k} \in \boldsymbol{\theta_v}$. But, in this case, the $\theta_{\cdot,k} \in \boldsymbol{\theta_v}$ over-represent opinions of vegans relative to non-vegans. This example shows that an important extension to the model would include co-variates at the level of the respondent and at the level of the item, not only because these are substantively meaningful, but because they can reduce distortions caused by the unequal amount of responses that we have from each respondent. Diagnostics and robustness will both be important areas of future research for models to estimate the opinion matrix from responses.

2 Computation

To make draws from this posterior distribution in equation 8 we use Markov chain Monte Carlo, specifically Gibbs sampling [9]. That is, we repeatedly draw from the conditional distribution for each parameter given the current values of the other parameters; for a review of Gibbs sampling, see [10]. However, before attempting to sample from this posterior distribution in this way, we perform two additional steps that greatly facilitate computation, but which do not affect the underlying model that we are estimating.

First, as described earlier, many respondents do not encounter many of the items. For example, in the voting data in Table 1, respondent 1 never encountered item 2 and respondent 2 never encountered item 1. Thus, as described earlier, there are actually two types of θ parameters, those that are informed by a specific vote (in this case, $\theta_{1,1}$, $\theta_{1,3}$, $\theta_{1,4}$, $\theta_{2,2}$, $\theta_{2,3}$, $\theta_{2,4}$) and those that are not (in this case, $\theta_{1,2}$, $\theta_{2,1}$). Thus, we note that

$$p(\boldsymbol{\theta} \mid \boldsymbol{Y}, \boldsymbol{X}, \boldsymbol{\mu}, \sigma) = p(\boldsymbol{\theta_v} \mid \boldsymbol{Y}, \dot{\boldsymbol{X}}, \boldsymbol{\mu}, \sigma) \times p(\boldsymbol{\theta_h} \mid \boldsymbol{\mu}, \sigma) \tag{6}$$

where $\boldsymbol{\theta_v}$ are parameters that are estimated from the votes and the hyper-parameters and $\boldsymbol{\theta_h}$ are parameters that depend on the votes only through the hyper-parameters, and $\dot{\boldsymbol{X}}$ is the reduced form of the original design matrix $\boldsymbol{X}$ that only includes columns for $\theta \in \boldsymbol{\theta_v}$. For example, for the

votes in Table 1, $\dot{\boldsymbol{X}}$ is

$$\dot{\boldsymbol{X}} = \begin{array}{c} \begin{array}{cccccc} \theta_{1,1} & \theta_{1,3} & \theta_{1,4} & \theta_{2,2} & \theta_{2,3} & \theta_{2,4} \end{array} \\ \left(\begin{array}{ccc|ccc} 1 & 0 & -1 & & & \\ -1 & 1 & 0 & & 0 & \\ 0 & -1 & 1 & & & \\ \hline & & & 0 & 1 & -1 \\ & 0 & & & & \\ & & & -1 & 0 & 1 \end{array}\right) \end{array}$$

In this simple example $\dot{\boldsymbol{X}}$ is 33% smaller than $\boldsymbol{X}$, but in both cases considered in the paper the reduction is much more substantial: $\dot{\boldsymbol{X}}$ is about 90% smaller than $\boldsymbol{X}$. Reducing the size of the design matrix in this way yields a substantial savings in terms of computing time and RAM needed to make draws from the posterior distribution. Given this fact, we can re-write equation 4 as follows:

$$\begin{aligned} p(\boldsymbol{\theta_v}, \boldsymbol{\theta_h} \mid \boldsymbol{Y}, \boldsymbol{X}, \boldsymbol{\mu}) \propto \quad & \left(\prod_{i=1}^{V} \Phi(\boldsymbol{x}_i^T \boldsymbol{\theta_v})^{y_i} (1 - \Phi(\boldsymbol{x}_i^T \boldsymbol{\theta_v}))^{1-y_i} \times \prod_{(j,k)}^{\theta_{j,k} \in \boldsymbol{\theta_v}} N(\theta_{j,k} \mid \mu_k, \sigma) \right) \\ & \times \left(1 \times \prod_{(j,k)}^{\theta_{j,k} \in \boldsymbol{\theta_h}} N(\theta_{j,k} \mid \mu_k, \sigma) \right) \end{aligned} \tag{7}$$

A second computational trick is to note that by introducing a latent variable $\boldsymbol{z}$ we are able to sample from the posterior more easily, an approach sometimes called data augmentation. Roughly, we are assuming that although we observe a discrete outcome y_i, there is actually an underlying continuous value z_i that generates y_i. As shown by Albert and Chib [11], including this continuous latent variable, z_i, in our model enables us to sample from the posterior distribution more easily. For a more thorough discussion of this type of data augmentation, see [12] and [13].

Combining these two computational tricks, we are left with the following posterior distribution:

$$
\begin{aligned}
&p(\boldsymbol{\theta_v}, \boldsymbol{\theta_h}, \boldsymbol{z}, \boldsymbol{\mu} \mid \boldsymbol{Y}, \dot{\boldsymbol{X}}, \sigma, \boldsymbol{\mu_0}, \boldsymbol{\tau_0^2}) \propto \\
&\quad \left(\prod_{v=1}^{V} (I(z_i > 0)I(y_i = 1) + I(z_i < 0)I(y_i = 0)) \times N(z_i \mid \dot{\boldsymbol{x}}_{\boldsymbol{i}}^{\boldsymbol{T}} \boldsymbol{\theta_v}, 1) \times \prod_{(j,k)}^{\theta_{j,k} \in \boldsymbol{\theta_v}} N(\theta_{j,k} \mid \mu_k, \sigma) \right) \\
&\quad \times \left(1 \times \prod_{(j,k)}^{\theta_{j,k} \in \boldsymbol{\theta_h}} N(\theta_{j,k} \mid \mu_k, \sigma) \right) \times \prod_{k=1}^{K} N\left(\mu_k \mid \mu_{0[k]}, \tau_{0[k]}^2\right) \qquad (8)
\end{aligned}
$$

In order to sample from the posterior distribution, we ran three parallel chains from over-dispersed starting values for 200,000 steps, saving every 200th draw, and discarded the first half of each chain as burn-in. At that point, all parameter estimates had approximately converged, $\hat{R} < 1.1$ [10], and so we combined the post burn-in draws to summarize the posterior distribution [14]. Overall, these computations took about 36 hours per dataset on a fast desktop computer.

The votes and ideas were then used to fit the model in equation 8 using Gibbs sampling with four update steps.

- Step 1: Draw $\boldsymbol{z} \mid \boldsymbol{Y}, \boldsymbol{\theta_v}, \dot{\boldsymbol{X}}$

Recall that $\boldsymbol{z}$ is the underlying latent outcome that we cannot observe. Based on ideas developed by Albert and Chib [11], we sample $\boldsymbol{z}$ from a truncated normal distribution such that $z_i > 0$ if $y_i = 1$ and $z_i < 0$ if $y_i = 0$. That is,

$$
z_i \sim \begin{cases} N(\dot{\boldsymbol{x}}_i^T \boldsymbol{\theta_v}, 1) I(z_i^* > 0) & \text{if } y_i = 1 \\ N(\dot{\boldsymbol{x}}_i^T \boldsymbol{\theta_v}, 1) I(z_i^* < 0) & \text{if } y_i = 0 \end{cases} \qquad (9)
$$

where I is an indication function which equals 1 when its argument is true and 0 when false [13]. This indicator function ensures that we are drawing from a properly truncated distribution. Computationally, we draw from the truncated normal using the `truncnorm` package in `R` [15].

- Step 2: Draw $\boldsymbol{\theta_v} \mid \boldsymbol{z}, \boldsymbol{\mu}, \dot{\boldsymbol{X}}, \sigma$

Under the data augmentation we used [11], once we have simulated $\boldsymbol{z}$, the latent outcome, we are left with a standard hierarchical linear model. To update $\boldsymbol{\theta_v}$ we use the "all-at-once" approach described in Gelman et al. [16].

That is,

$$\boldsymbol{\theta_v} \sim N(\hat{\boldsymbol{\theta}}_{\boldsymbol{d}}, \hat{\boldsymbol{V}}_{\boldsymbol{\theta_v}}) \tag{10}$$

where

$$\hat{\boldsymbol{\theta}}_{\boldsymbol{d}} = (\widetilde{\boldsymbol{X}}^T \widetilde{\boldsymbol{\Sigma}}^{-1} \widetilde{\boldsymbol{X}})^{-1} \widetilde{\boldsymbol{X}}^T \widetilde{\boldsymbol{\Sigma}}^{-1} \widetilde{\boldsymbol{Y}} \quad , \quad \hat{\boldsymbol{V}}_{\boldsymbol{\theta_v}} = (\widetilde{\boldsymbol{X}}^T \widetilde{\boldsymbol{\Sigma}}^{-1} \widetilde{\boldsymbol{X}})^{-1}$$

$$\widetilde{\boldsymbol{X}} = \begin{pmatrix} \dot{\boldsymbol{X}} \\ \boldsymbol{I} \end{pmatrix} \quad , \quad \widetilde{\boldsymbol{Y}} = \begin{pmatrix} \boldsymbol{Y} \\ \tilde{\boldsymbol{\mu}} \end{pmatrix} \quad \text{, and} \quad \widetilde{\boldsymbol{\Sigma}} = \begin{pmatrix} \boldsymbol{\Sigma_y} & \boldsymbol{0} \\ \boldsymbol{0} & \boldsymbol{\Sigma_\theta} \end{pmatrix}.$$

Further, $\boldsymbol{I}$ is the identity matrix, $\boldsymbol{\Sigma_y} = \text{Diag}(1)$, $\boldsymbol{\Sigma_\theta} = \text{Diag}(\sigma)$, and $\widetilde{\boldsymbol{\mu}}$ is a vector that is the same length as $\boldsymbol{\theta_v}$ and represents an "expanded" version of $\boldsymbol{\mu}$. That is, if the ith column of $\dot{\boldsymbol{X}}$ represents item k (independent of what respondent is involved), then the ith element of $\widetilde{\boldsymbol{\mu}}$ is μ_k.

Computationally, we note that $\widetilde{\boldsymbol{X}}$ and $\widetilde{\boldsymbol{\Sigma}}$ are almost all zeros, so the calculations described above to make a draw are made using sparse matrix routines that are implemented in the `Matrix` package in `R` [17].

- Step 3: Update $\boldsymbol{\theta_h} \mid \boldsymbol{\mu}, \sigma$

A large number of the θ parameters are determined by data only through the hyper-parameters. For these θ, which we call $\boldsymbol{\theta_h}$, we update as follows:

$$\theta_{j,k} \sim N(\mu_k, \sigma) \quad \forall \quad \theta_{j,k} \in \boldsymbol{\theta_h} \tag{11}$$

Thus, this step is roughly like an imputation based on the overall estimated characteristics of the population. Computationally, no special steps are required to make these updates.

- **Step 4:** Update $\boldsymbol{\mu} \mid \boldsymbol{\theta_v}, \boldsymbol{\theta_h}, \sigma, \boldsymbol{\mu_0}, \boldsymbol{\tau_0^2}$

$$\mu_k \sim N(\mu, \tau^2) \tag{12}$$

where

$$\mu = \frac{\frac{1}{\tau_0^2}\mu_0 + \frac{n}{\sigma^2}\bar{\theta}_{\cdot,k}}{\frac{1}{\tau_0^2} + \frac{n}{\sigma^2}} \quad \text{and} \quad \tau^2 = \frac{1}{\frac{1}{\tau_0^2} + \frac{n}{\sigma^2}}$$

where $\bar{\theta}_{\cdot,k}$ is the mean of the θ for a specific item k (that is, $\frac{1}{J}\sum_{j=1}^{J}\theta_{j,k}$) and n is the number of estimates involved (in this case, the number of user-sessions, J). See [18, Ch. 6] for a derivation. No special computational issues are involved in this update.

3 Data processing

[1]

In order to analyze the data collected in these two case studies, we followed a three-step procedure. First, using the standard features available to any wiki survey creator at `www.allourideas.org`, we downloaded comma-separated value (csv) files that record respondent activity in that wiki survey. Second, we cleaned the csv files to correct for website errors that occurred during data collection. More specifically, there were two main data cleaning steps caused by website errors: 1) for a small fraction of participants, `www.allourideas.org` automatically created a new session after each vote; and 2) for participants whose sessions timed out after 10 minutes (see SI 1, Sec 3 for more on sessions and session time-outs), `www.allourideas.org` improperly assigned some information to the old session instead of the new session. After these website issues were discovered while writing this paper, we have improved the code at `www.allourideas.org` so that these problems no longer occur. Finally, after cleaning, we subset the data so that we only estimated parameters for items with at least one win from a valid vote and at least one loss from a valid vote. For completeness, we describe, in detail, the changes that took place between the data we downloaded from the website and the data that we used for estimation.

3.1 PlaNYC

The raw data files from the website included 489 ideas—25 seed ideas and 464 user-contributed ideas—as well as 31,893 responses—26,727 valid votes, 1,988 invalid votes, and 3,178 skips—from 2,094 sessions. There were no responses or ideas uploaded outside of the appropriate time window of 2010-10-07 to 2011-01-30.

Cleaning the files caused two main changes. First, because of participants who were erroneously placed in a new session after each vote, 52 actual sessions had originally been misrepresented as 710 sessions. Therefore, we collapsed these 710 sessions to the appropriate 52 sessions. This change in session definitions created 30 valid votes that were immediately preceded by a skip, so we invalidated these 30 votes (see SI 1, Sec 3 for more on invalidating votes after skips to improve

[1]In this case, the number of user-contributed ideas per session is somewhat difficult to interpret because some user-contributed ideas were bulk-uploaded by the Mayor's Office following community meetings at which ideas were recorded on paper. Unfortunately no records were kept of this bulk uploading, so we cannot distinguish it from other respondent behavior.

data quality). Then, after correcting for errors caused by session time-outs, 9 valid votes were immediately preceded by skips, so we invalidated these 9 votes. Thus, after cleaning, the files contained 489 ideas—25 seed ideas and 464 user-contributed ideas—as well as 31,893 responses—26,688 valid votes, 2,027 invalid votes, and 3,178 skips—from 1,436 sessions.

When applying our model to the case studies in this paper, we estimated parameters for all items that were active on the final day and had at least one valid win and at least one valid loss. In PlaNYC there were 269 such items with 26,604 valid votes among them, cast from 1,397 sessions. Thus, for PlaNYC the opinion matrix, $\boldsymbol{\theta}$, had dimension 1,397 $\times$ 269.

3.1.1 OECD

The raw data files from the website included 594 ideas—35 seed ideas and 559 user-contributed ideas—as well as 30,763 responses—27,133 valid votes, 1,338 invalid votes, and 2,292 skips—from 3,373 sessions. The OECD conducted a period of internal pilot testing from 2010-09-03 to 2010-09-15, and we dropped the 1,747 votes and 164 skips contributed from 182 sessions during this time. We also converted the 25 ideas contributed during the internal pilot testing to seed ideas. No responses or ideas were contributed after 2010-10-15.

Cleaning the files caused two main changes. First, because of participants who were erroneously placed in a new session after each vote, 104 actual sessions had originally been represented as 1,627 sessions. Therefore, we collapsed these 1,627 sessions to the appropriate 104 sessions. This change in session definitions created 93 valid votes that were immediately preceded by a skip, so we invalidated these 93 votes. Then, after correcting for errors caused by session time-outs, 8 valid votes were immediately preceded by skips, so we invalidated these 8 votes. Thus, after cleaning, the files contained 594 ideas—60 seed ideas and 534 user-contributed ideas—as well as 28,852 responses—25,393 valid votes, 1,331 invalid votes, and 2,128 skips—from 1,668 sessions.

When applying our model to the case studies in this paper, we estimated parameters for all items that were active on the final day and had at least one valid win and at least one valid loss. In OECD there were 285 such items with 23,845 valid votes among them, cast from 1,620 sessions. Thus, for OECD the opinion matrix, $\boldsymbol{\theta}$, had dimension 1,620 $\times$ 285.

References

1. Thurstone LL. The Method of Paired Comparisons for Social Values. Journal of Abnormal and Social Psychology. 1927;21(4):384–400. Available from: http://www.sciencedirect.com/science/article/B8H4H-4NRF2VD-7/2/2788af4395b6179abd589678c1308a07.

2. Mosteller F. Remarks on the Method of Paired Comparisons: I. {The} Least Squares Solution Assuming Equal Standard Deviations and Equal Correlations. Psychometrika. 1951 Mar;16:3–9. Available from: http://www.springerlink.com/content/58p1h351uq196180/.

3. Bradley RA, Terry ME. Rank Analysis of Incomplete Block Designs: I. {T}he Method of Paired Comparisons. Biometrika. 1952 Dec;39(3/4):324–345. Available from: http://www.jstor.org/stable/2334029.

4. Stern H. A Continuum of Paired Comparisons Models. Biometrika. 1990 Jun;77(2):265–273. Available from: http://biomet.oxfordjournals.org/content/77/2/265.abstract.

5. Stern H. Are All Linear Paired Comparison Models Empirically Equivalent? Mathematical Social Sciences. 1992;23(1):103–117.

6. Gelman A, Hill J. Data Analysis Using Regression and Multilevel/Hierarchical Models. New York: Cambridge University Press; 2006.

7. Rossi PE, Allenby GM, McCulloch R. Bayesian Statistics and Marketing. Hoboken, NJ: Wiley; 2006.

8. Train KE. Discrete Choice Methods with Simulation. 2nd ed. Cambridge: Cambridge University Press; 2009.

9. Gelfand AE, Smith AFM. Sampling-Based Approaches to Calculating Marginal Densities. Journal of the American Statistical Association. 1990 Jun;85(410):398–409. Available from: http://www.jstor.org/stable/2289776.

10. Gelman A, Carlin JB, Stern HS, Rubin DB. Bayesian data analysis. 2nd ed. Boca Raton: Chapman and Hall/CRC; 2003.

11. Albert JH, Chib S. Bayesian Analysis of Binary and Polychotomous Response Data. Journal of the American Statistical Association. 1993 Jun;88(422):669–679. Available from: http://www.jstor.org/stable/2290350.

12. Lynch SM. Introduction to Applied Bayesian Statistics and Estimation for Social Scientists. New York: Springer; 2007.

13. Jackman S. Bayesian Analysis for the Social Sciences. New York: Wiley; 2009.

14. Gelman A, Shirley K. Inference from Simulations and Monitoring Convergence. In: Brooks S, Gelman A, Jones G, Meng XL, editors. Handbook of Markov Chain Monte Carlo. Boca Raton: CRC Press; 2011. p. 163–174.

15. Trautmann H, Steuer D, Mersmann O, Bornkamp B. truncnorm: Truncated Normal Distribution; 2011. R package, version 1.0-5.

16. Gelman A, van Dyk DA, Huang Z, Boscardin JW. Using Redundant Parameterizations to Fit Hierarchical Models. Journal of Computational and Graphical Statistics. 2008 Mar;17(1):95–122. Available from: http://pubs.amstat.org/doi/abs/10.1198/106186008X287337?journalCode=jcgs.

17. Bates D, Maechler M. Matrix: Sparse and Dense Matrix Classes and Methods; 2011. R package version 0.999375-50.

18. Hoff PD. A First Course in Bayesian Statistical Methods. 2nd ed. Dordrecht: Springer; 2009.
